# Perforator Based Propeller Flaps in Limb Reconstructive Surgery: Clinical Application and Literature Review

**DOI:** 10.1155/2014/690649

**Published:** 2014-08-27

**Authors:** Stefano Artiaco, Bruno Battiston, Giulia Colzani, Pasquale Bianchi, Gabriele Scaravilli, Elena Boux, Pierluigi Tos

**Affiliations:** ^1^Department of Orthopaedics, Traumatology and Rehabilitation, Microsurgery Unit, Azienda Ospedaliera Universitaria, Città della Salute e della Scienza, Orthopaedic and Trauma Center, Via Zuretti 29, 10126 Turin, Italy; ^2^Department of Orthopaedics, Traumatology and Rehabilitation, Orthopaedic Clinic, Second University of Naples, Via S. Andrea Delle Dame, 80100 Naples, Italy

## Abstract

The reconstruction of loss of substance due to trauma or oncological excision may have relevant functional and aesthetic implications. We report our experience in twenty-one cases of propeller flaps for the treatment of loss of substance of the upper and lower limbs. The etiology of defect was tumor excision in nine cases, trauma in seven cases, surgical wound complications in four cases, and chronic osteomyelitis in one case. Clinical results were favorable in most cases and eighteen flaps survived. We observed an overall complication rate of 33% with four cases of superficial epidermolysis that spontaneously healed and three cases of partial flap loss ranging from 10 to 50% that required surgical revision by means of skin graft (two cases) or ALT free flap (one case). Propeller flap harvesting requires great care and experience, and potential complications may occur even in expert hands. When indicated by the characteristic of the defect, these flaps can be a useful surgical option for the treatment of loss of substance of upper and lower limbs.

## 1. Introduction

The reconstruction of loss of substance due to trauma or oncological excisions has relevant functional and aesthetic implications. Some kind of flaps used for the treatment of upper and lower limb lesions required the sacrifice of major vascular bundles. During the last decades, anatomical studies on skin vascularization provided the base for the development of flaps nourished by perforating arteries and preserving major vascular axis [1–4].

According to the definition established during the Consensus Conference of Gent in 2003, perforator flaps are constituted by cutaneous and subcutaneous tissue areas nourished by perforator arterial branches originating from major vascular bundles with an intramuscular or intraseptal course. Based on experimental studies, Taylor et al. reported that a single perforator may safely supply its proper angiosome and up to the half of vascular territory of the adjacent perforator [5, 6]. This possibility is favored by vascular adoption directed toward periphery that occurs by means of increased vascular pressure in the perforator artery after ligature of collateral subcutaneous and intramuscular arterial branches. One of the main characteristics of perforator flaps is their versatility. The flap can be selected on the perforator artery depending on the size and the location of defect and can be used both as a free or local flap, exploiting the possibility of advancement or twisting of the vascular pedicle. In case of coverage by means of a V-Y type advancement, local perforator flaps can reach considerable displacements covering distances greater than those obtained by the use of standard V-Y flaps.

The propeller flap represents a model of local perforator flap that was first described by Hyakusoku et al. for the treatment of periarticular skin retractions secondary to burns around the elbow [7]. In this model the flap is harvested around a cutaneous perforator arterial branch by twisting of the vascular pedicle and rotating the skin paddle like a propeller up to a maximum angle of 180°. The blades of the propeller flap are prepared according to the type of defect under direct observation of the origin and direction of the perforating vessels of the island. The pedicle can be isolated by means of loupes and microscope is normally not required [8, 9]. Therefore, as reported by Georgescu et al., this local perforator flap that requires a microsurgical dissection without vascular sutures can be defined as a “microsurgical not microvascular flap” [10]. The absence of vascular sutures and the preservation of major vascular and underlying muscles are the main advantages of propeller flaps. If required and if technically possible, the local perforator flap can be adapted to the reconstructive needs preparing composite flaps that include muscular, tendinous, and skeletal components. Moreover, from the aesthetic point of view, the reconstruction of the defect can be achieved with optimal results as it takes into account the concept of like-with-like reconstruction by means of donor areas close to that of the defect.

Thanks to these potential benefits, the use of local perforator flaps is constantly increased in clinical practice over the time. The aim of this study is to report our clinical experience and results with propeller flap in reconstructive surgery of upper and lower extremities updating our initial case series [11]. Further we review current indications and surgical questions regarding such kind of flaps.

## 2. Materials and Methods

Our study included twenty-one patients operated on between 2006 and 2013 in our Department of Reconstructive Microsurgery by means of perforator-based propeller flaps for the reconstruction of loss of substance of limbs secondary to trauma or tumor excision.

Thirteen patients were male and eight patients were female. Age ranged from 22 to 86 years (mean 54.5).

In seven cases the defect was located in the upper limb and in fourteen cases in the lower limb.

Concerning the site, in the upper limb, the defect was at the elbow in two cases, on the dorsal aspect of the hand in three cases, and at the finger level in two cases; in the lower limb the defect was at thigh in three cases and at leg or ankle in eleven cases.

The etiology of defect was tumor excision in nine cases, trauma in seven cases, surgical wound complications in four cases, and chronic osteomyelitis in one case.

The perforator vessel originated in the upper limb from radial artery in three cases, dorsal metacarpal artery in two cases, and superior ulnar collateral artery in two cases; in the lower limb the perforator vessel originated from posterior tibial artery in seven cases, peroneal artery in three cases, vastus lateralis in two cases, genicular artery in one case, and anterior tibial artery in one case.

The size of defect ranged from 7 × 8 cm to 1 × 5 cm in the upper limb (mean 21 cm^2^) and from 25 × 15 cm to 10 × 3 cm (mean 138.5 cm^2^) in the lower limb.

## 3. Surgical Technique

**Figure 1:**
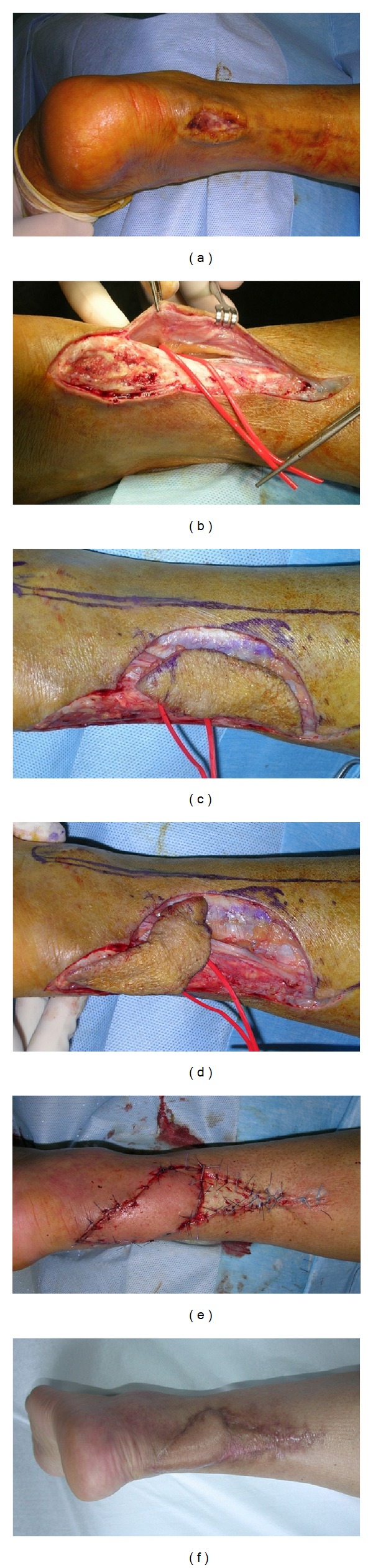
(a) 70-year-old BR male, diabetic. Postsurgical wound defect after Achilles tendon repair. (b) Propeller flap based on peroneal artery perforator vessel. (c) Flap harvested and perfusion checked. (d) Flap 160° rotated on defect area. (e) Closure with skin graft of donor area. (f) Final clinical result.

An ultrasound Doppler scanner was used before operation in order to detect perforator arteries in donor site area [12]. On this base the flap was planned considering position and dimension of the defect and the need to avoid tension on the edges of propeller flap during suture. The procedures were performed under magnification loupes (2.5–4.0x) with microsurgical instruments and technique. After skin incision, direct visualization of perforator vessels was performed by means of subfascial approach [13, 14] and the perforator vessel was chosen before flap harvesting taking into account the size of pedicle and the distance from the recipient site (Figures 1(a)-1(b)). The flap was then harvested with the rotation center situated on the emergency point of perforator artery (Figures 1(c)-1(d)). The fascia was included into the flap according to characteristics of donor site area and of the defect such as in the case of bone exposure. The dissection of vascular pedicle was a fundamental point of surgical technique. It was necessary to (a) isolate with blunt dissection the pedicle at least for 1.5 cm and (b) avoid pedicle stretching during flap harvesting and positioning. Flap perfusion was checked before rotation waiting few minutes and irrigating skin paddle with warm saline solution in order to enhance microvascular dilatation. When possible, after flap rotation, direct closure of the donor site without tension was performed. Skin graft was used in some cases to complete closure avoiding tensioning and vascular straining in the propeller flap (Figure 1(e)). Drains were usually removed 24 hours after surgical procedure. The limb was elevated and soft bandage was applied avoiding compression leaving a noncovered area in order to check skin color and temperature. Splinting for about two weeks was indicated when a skin graft was used. Low weight molecular heparin was administered when weight bearing was not allowed.

## 4. Results

Eighteen out of twenty-one patients healed with complete flap survival. In this group four patients (19%) showed superficial epidermolysis with spontaneous resolution and secondary healing of the flaps. These flaps were located in the upper limb in one case (superior ulnar collateral artery) and in the lower limb in three cases (vastus lateralis propeller flap in two cases; posterior tibial artery propeller flap in one case).

**Figure 2:**
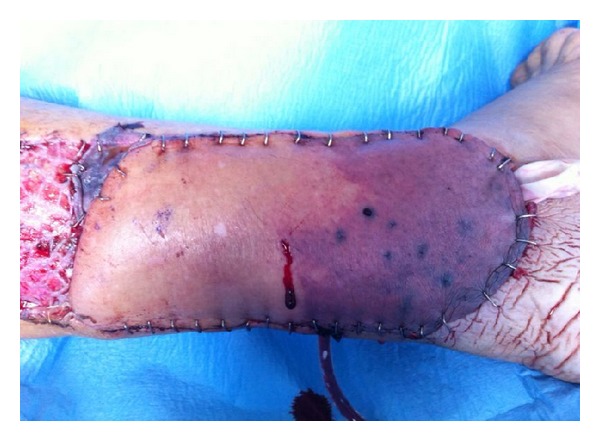
Propeller flap based on posterior tibial artery. Clinical view of partial flap necrosis.

Three patients (14%) showed a partial necrosis involving the propeller flap. The rate of flap necrosis ranged from 10 to 50%. One patient with a genicular artery based propeller flap with 10% of flap necrosis and one patient with a posterior tibial artery based propeller flap with 40% flap necrosis were treated by means of skin graft. One patient with a posterior tibial artery based propeller flap with 50% flap necrosis (Figure 2) was treated by means of that required a reconstruction by means of a free anterolateral thigh (ALT) flap. The overall complication rate was 33% (upper limb 14%; lower limb 42%).

## 5. Discussion 

The treatment of simple and complex loss of substance should ideally allow reconstruction with like-with-like tissues preserving major vascular axis and minimizing donor site pathology, operative time, and hospitalization time. The vascularization of the skin has been the topic of several anatomical researches that allowed the development of reconstructive techniques based on local flap nourished by cutaneous perforator arteries.

The perforator-based propeller flaps, harvested around a perforator pedicle by means of the rotation of skin paddle, are now currently used in clinical practice in order to cover loss of substance of upper and lower extremities. Basic concepts, principles of surgical technique, guidelines, and indications of perforator-based propeller flaps are nowadays well established [8, 9]. 

*Principles of Propeller Flap Surgery*

- Preoperative evaluation and planning of the flap:
   careful evaluation of patient history and etiology of defect;
   identification of potential surgical risk factors;
   identification of size and location of loss of substance;
   identification of surrounding perforator vessels by ultrasound Doppler and provisional flap design including a propeller blade area of 1.0 × 0.5 cm larger than the original defect area.
- Surgical technique:
   careful subfascial dissection and direct visualization of perforator vessels (optical magnification device);
   careful dissection of the vascular pedicle for an adequate length and flap harvesting (optical magnification device);
   check of flap perfusion (irrigation with warm saline solution) and rotation of the flap;
   direct closure of the defect or additional skin graft when direct closure is not possible.
- Postoperative care:
   clinical observation of propeller flap checking skin color and temperature;
   drain removal at 24–48 hours post-op;
   immobilization by splinting in case of skin graft application;
   low weight heparin administration in case of nonweight bearing.

Clinical experience with propeller flaps for the treatment of loss of substance of the limbs has constantly increased during the last decade and included repair of trauma-induced injuries, posttrauma revision, tumor resection, chronic infection, pressure sores, and chronic ulcers. Recent systematic reviews showed that, considering major clinical series, about ninety-nine cases of upper limb defects and one hundred eighty-six cases of lower limb defects treated by means of propeller flaps have been reported in the literature [15, 16].

In the upper limb the reconstruction of loss of substance with propeller flap may offer, together with functional recovery, an optimal aesthetic result due to a like-with-like tissue repair. At the arm and elbow the propeller flaps harvesting is facilitated by the length of the vascular pedicles, while at the forearm the relative shortness of perforator branch makes an adequate release for twisting the skin paddle difficult. When the use of propeller flaps is not possible due to technical limits, the coverage of the defect should then be performed by means of perforator-based flaps with fascial pedicle or by local flaps with sacrifice of a major vascular bundle. The clinical experiences reported in the literatures [10, 17, 18] demonstrated that the propeller flaps can be employed with positive results in the upper limb for the treatment of traumatic loss of substances, as well as after tumor excisions, burns, and other conditions. In selected cases even complex defects may be treated with propeller flaps harvested as composite flaps. We reported a successful case of reconstruction of the dorsal aspect of the index finger with extensor tendon loss by means of a composite propeller flap 180° rotated and based on dorsal metacarpal artery including the* extensor proprius indicis* tendon in order to restore continuity of extensor common tendon of the index finger [19]. As regards complications, some authors did not observe problems in their clinical series [17, 18]. Venous congestion and partial flap loss were instead reported in a small number of patients by other surgeons [20–22].

In the lower limb the perforator-based propeller flaps may represent a useful solution for the coverage of the defects in the thigh and knee area, in internal and external malleolar areas, and in the heel and Achilles tendon region [8, 9]. These flaps are particularly reliable in the distal leg and at the ankle region where the defects are often small but difficult to treat by means of other local flaps. In such cases the propeller flaps may be an alternative to free flaps which have been traditionally used in clinical practice.

In some conditions the inadequate length of the perforating vessels in the region of the leg and ankle can interfere with the transposition of the flap [23]. Despite this, many authors reported clinical series using propeller flaps for the reconstruction of the lower limb [11, 24–26].

The systematic review of the literature showed that in most cases the results are favorable and that propeller-based perforator flaps appeared to be a safe and reliable procedure for the coverage of soft tissue defects of the lower limb [16]. Furthermore, it has been shown that the overall complication rate of propeller flaps in lower limb was 25.8% with a failure rate of 1.1% and that the most common complications were partial flap loss and venous congestion (11.3% and 8.1%) [16]. Similar results were reported by Innocenti who observed an overall complication rate of 44% in lower limb (twenty-eight out of sixty-six flaps) [27]. Eighteen out of the twenty-eight complications (64%) healed with no further treatment; eight patients underwent skin grafting and one patient each experienced total flap failure (2%) and partial flap failure (2%). In this study any specific risk factor related to complications such as arc of rotation, flap size, age, sex, smoking, defect etiology, diabetes, and peripheral vascular diseases was not identified [27].

Minor common complications, such as transient edema, epidermolysis, or partial flap necrosis, may be conservatively managed in most cases. Flap failure, even if rare, is the most fearful complication because it may cause a defect larger than the initial loss of substance and is difficult to treat. It has been hypothesized that the risk of partial necrosis and flap failure could be related to the dimension of skin area included in the flap. This problem is currently object of discussion in the light of recent knowledge regarding the vascularization of skin area nourished by perforator vessels named perforasomas [4]. Implications of this theory are relevant and may eventually help to improve surgical technique and clinical results of propeller flaps. Despite potential complications these flaps should be considered among the possible reconstructive surgical options both in upper and in lower limbs defects. Nonetheless, free flaps still remain a mandatory choice for covering wide cutaneous area and for the reconstruction of complex defects requiring composite or functional flaps.
